# Supplementary material for: Anterior CNS expansion driven by brain transcription factors
Source: eLife. 2019 Jul 4;8:e45274. doi: 10.7554/eLife.45274 (PMC6634974; doi:10.7554/eLife.45274)
Supplement: Figure 1—source data 1. [file elife-45274-fig1-data1.docx]

**Figure 1-source data 1**

**gRNA and deleted sequences for CRISPR/Cas9 deletion of *Rx*, *otp* and *hbn***

***Rx***

Forward: CTTCCACTTCGCGCCATCCC CGG input + strand [2R:20922100…20922122](http://flybase.org/cgi-bin/gbrowse/dmel6/?name=2R%3A20922100..20922122)

Revcomp: CGCGGGAATAGACATCGGGG AGG input + strand [2R:20923858…20923880](http://flybase.org/cgi-bin/gbrowse/dmel6/?name=2R%3A20923858..20923880)

***otp***

Forward: CAAGCCAATTGATGCATGAC AGG input + strand [2R:20,895,521…20,895,543](http://flybase.org/cgi-bin/gbrowse/dmel6/?name=2R%3A20895521..20895543)

Revcomp: ACGGCCTTTTTAGACGCACT CGG input - strand [2R:20,896,874...20,896,896](http://flybase.org/cgi-bin/gbrowse/dmel6/?name=2R%3A20896874..20896896)

***hbn***

Forward: TTAGTTCG|TTAGTCGGAGGA GGG input - strand [2R:20957979..20958001](http://flybase.org/cgi-bin/gbrowse/dmel6/?name=2R%3A20957979..20958001)

Revcomp : AACGAGAGGACTCCCCAACG TGG input + strand [2R:20958643..20958665](http://flybase.org/cgi-bin/gbrowse/dmel6/?name=2R%3A20958643..20958665)

***Rx^ST1^* mutant deleted sequences**

Green: primers #5 to amplify the region of the deletion

Light blue: gRNA location

Red: deleted sequence

TCTCGAGCAGCAACTTCCCAGTCTGACTGCGTAATTGGTTTTGTTTCCA

GTTGGCCCGCCCCGAAAGTCAATTTAAGCATAGAAATTGAATTCGGTTA

CACAAAATCCCAGCCAAGAACGGCCAAAGAACTGAAACCTAAACCCCAA

ATAATATATAAAGCTTGGCCTCGGCGCTTCGAAAACTAATGACTTCCTT

GCATGTCACGTGATATGAACAAGCGCTAGTTGGAACACCCATTAGGGAC

CAATGCCGCCTCATCTGGAACTCCTCCTCTGCTTTCTGGGAATTTTTAT

TGCCTCACATTGTAAATACCTGGCGTCTAATTGAAAGCCGTTTATTAGT

GGGTGGGCGGCAGTCGGCCAACAGGTTTTGACCCGCCGCAGTTCGAAGG

CTTCCACTTCGCGCCATCCCCGGTTCAGTGGGCAAATTAACTAACACGT

TGTTATTAGCCGGGTTGTTCAAGGCTTTTAGCCAGCGAGTTGGCAAACA

GCTTTATGACTTACCTACCCGCCCCATTGAGCATACTAATTAAAATCTA

ATCAGGTGCCTGAATGCGTGGGCGAATGGAAAAGGAAGCTCCAACCCCA

TTGCCCCGTATTAGCCATTGATCACGCTCAATTAATTGCTCAAAAAAAT

AATGATTGCTATTCCAGGATTCCCTTGTAAAGTAGCTATTATTACTCCT

TGACATATATCCTTTATTAACACTACAAACTTCATATTTCCTGTAACTT

TTGATCTCGCTTTTAGGCTCCGTTGATCCCTCCCTGGGTGACGATGATG

CCACTGATCTACGCTGCGGCATGACCCTGACGCAGTTGCGCAGCATGGA

CAATCACATGGCCAGTATGCTGCAGCAGCACGCGAAAAACGGCGGAGCC

CTGCCCTACGGTCCACCAACTCCGCCGGGGGGTCAGCAGCCCCAGGTGC

CGAATGCCACGCCCCTGCACCATGGACAACAGATGGGCGGCCAGGCGGG

ACATGCAACACATGCCGGACATGGTCATCCTACGCATCATGGGCATGCG

CCCTTCGGCTATCATAATGCATTTGGCTTTGGCCAGGGCGGTCATGGGT

ATGGGCATCCGGAGGAGGCGGCGGGCAATTACCTCAATTCGATGCACCA

GATGGTCGAGGCCAATCAATTGCAGACCGGAGCCAATGGGGCCAATCCA

CCGCCCGCTCCGCTGCCGCCCAGTTCCTTTGGCAGTCATCAGCAGCATT

TGGCCGCCTTGGCGGCTCAGGCGCAGGAGCAGCAGAATCAACACAGCAA

ATACGCCAAGTCATCGCCCACTGGAGCGGGTCCTCCTCCACCGCCGGGT

GCCTACTTTATGGAATCCCAGACGGCGCCTGTCGCCCCGTCGCAAATCA

ACTACGACGAGCGTTCAATGTCCTCGGCCAGCGATTTGGAGGAGGATGA

CGATGATGCGGCAAAGTTGCAGCTCGATGTGACCTCGCCGCCCACTCCG

TCGCCACGTGGTCAGCTGGCCGCCAAGCGCAAGTCCGCCGGCGTCTTCT

GTGATGATAATGAGCCAAAATTAGCCAACGGTCAGTTGCCAGGCGGCAA

TTACGGCATTCGTCCGCGCAGCATGGAGGAGGTGCATCATCAGCAGCAA

TCGCATCACCATCAACAACAACAGCAGCAGCAGCAACAGCAACAGCAAC

TGCAACAGCAGCAGGGCTTTCAGCACGACTTTCGCAACAGTGGCAATGG

GAATCCCAATGGCAACAGCAATTCCGGCGATCATGGCGAGCGTTTGAAC

GCGGATAGCGACAGCTTGGTCAACGGCAGCTGTGCCTCCAGCGAGGATC

TCAACCAGACCAACAGCAGCGAGCAGGGCGAGAAGATCACCTCCGGCAG

CGACGACGAAGGTGGGTACAACGTCTATCCTTTAATGTCCTTTTGATAA

AAGAAAGCATAATGCTGATAACTTACTAGCTTAATCATCGATCAAGTTG

TTTTATTGGTAATAAGATACCAGTATCTTAACGTATCTTATTCACACCT

TTAACAAACATCTGTATTGCAATTTTTTGTATTGCCAGGTCAAGATGAC

AACTGCGCCAAGAAGAAACACCGTCGCAATCGGACCACCTTCACCACCT

ACCAGCTCCATGAGCTGGAGCGGGCCTTCGAGAAGTCCCACTACCCCGA TGTCTATTCCCGCGAGGAGCTGGCCATGAAGGTGAACCTGCCCGAGGTC

CGCGTCCAGGTGAGTCACCTCCACCAGTACCCACCAATATCTCCCGTAT

CTCTTGTGTCTGGTAGTGATTTACATTCGAGTCTCGGCTAATTGGGAAA

TCAACGTGTCTGCAGCTGGAG

***otp^ST1^* mutant sequences**

Deletion at forward site

Underline: gRNA

Red: deleted sequence in genomic DNA (determined by PCR, cloning and Sanger sequencing)

CAAGCCAATTGATGCATGAC AGG input + strand [2R:20,895,521…20,895,543](http://flybase.org/cgi-bin/gbrowse/dmel6/?name=2R%3A20895521..20895543)

Deletion at reverse site

Underline: gRNA

Blue: genomic target region

Red: mutated/inserted sequence (determined by Illumina whole genome sequencing)

ACGGCCTTTTTAGACGCACT CGG [2R:20,896,874...20,896,896](http://flybase.org/cgi-bin/gbrowse/dmel6/?name=2R%3A20896874..20896896)

ACGGCCTTTTT-------------------------AGACGCACT [2R:20,896,874...20,896,896](http://flybase.org/cgi-bin/gbrowse/dmel6/?name=2R%3A20896874..20896896)

ACGGTCATGTTCACCAGGTCACCAGGCGCACT
