# Supplementary material for: Anterior CNS expansion driven by brain transcription factors
Source: eLife. 2019 Jul 4;8:e45274. doi: 10.7554/eLife.45274 (PMC6634974; doi:10.7554/eLife.45274)
Supplement: Figure 2—source data 1. [file elife-45274-fig2-data1.docx]

**Figure 2-source data 1**

|  | **elav-Gal4/+ (control)** | | **elav>UAS-erm,-tll** | | **elav>UAS-Tetra** | |
| --- | --- | --- | --- | --- | --- | --- |
|  | **thorax** | **abdomen** | **thorax** | **abdomen** | **thorax** | **abdomen** |
| Average cell volume (µm^3^) | 36,07 | 31,16 | 36,97 | 36,16 | 36,46 | 35,05 |
| SD | 6,01 | 3,88 | 10,60 | 5,45 | 4,95 | 12,28 |
| **Average cell volume VNC (T+A)** (µm^3^) | **33,39** |  | **36,57** |  | **35,75** |  |
| SD VNC (T+A) | 5,34 |  | 8,04 |  | 8,96 |  |

**Estimated average cell volume per region and genotype at AFT**

The values for average cell volume of VNC were used to estimate total cell numbers for the three respective genotypes at AFT (Fig. 2D). The ratio of DAPI volume of a restricted region in thorax or abdomen to the total number of cells counted manually in the same region was used as a measure of the average cell volume. Independent measurements were done in thoracic and abdominal regions of the VNC for each genotype. No significant difference was observed between values measured in thorax and abdomen for each genotype (two-tailed Student’s T-test, n≥3 embryos, n≥5 measurements per region and genotype). Neither between all thoracic and abdominal measurements across the three genotypes (two-tailed Student’s T-test, p=0.355, n≥9 embryos, n≥17 measurements per region). Since no difference was found for cell volumes between thorax and abdomen for each genotype, the average of all measurements was used as the average cell volume for VNC per genotype to calculate total cell number for each respective genotype. No significant difference was found across the three genotypes irrespective of the region (one-way ANOVA, p=0.596, n≥3 embryos, n≥11 measurements per genotype, Bonferroni Post Hoc test). SD= standard deviation.
